# Human Multidrug-Resistant *Salmonella* Newport Infections, Wisconsin, 2003–2005

**DOI:** 10.3201/eid1311.061138

**Published:** 2007-11

**Authors:** Amy E. Karon, John R. Archer, Mark J. Sotir, Timothy A. Monson, James J. Kazmierczak

**Affiliations:** * University of Wisconsin School of Medicine and Public Health, Madison, Wisconsin, USA; † Wisconsin Division of Public Health, Madison, Wisconsin, USA; ‡ Wisconsin State Laboratory of Hygiene, Madison, Wisconsin, USA; 1 Current affiliation: Centers for Disease Control and Prevention, Atlanta, Georgia, USA

**Keywords:** Agriculture, cattle, ceftriaxone, drug-resistance, multiple, bacterial, dairy products, salmonella, dispatch

## Abstract

We conducted a retrospective study of *Salmonella* Newport infections among Wisconsin residents during 2003–2005. Multidrug resistance prevalence was substantially greater in Wisconsin than elsewhere in the United States. Persons with multidrug-resistant infections were more likely than persons with susceptible infections to report exposure to cattle, farms, and unpasteurized milk.

During the past decade, multidrug-resistant (MDR) *Salmonella* *enterica* serotype Newport strains in the United States have increased substantially (*1*). The prevalence of the most common MDR *S.* Newport phenotype, Newport-MDRAmpC, increased from 1% of human *S.* Newport isolates tested in 1998 to 21% of isolates tested in 2003 (*2*). Newport-MDRAmpC is resistant to at least chloramphenicol, streptomycin, sulfamethoxazole/sulfisoxazole, tetracycline, amoxicillin-clavulanic acid, ampicillin, cefoxitin, ceftiofur, and cephalothin. This phenotype exhibits decreased susceptibility to ceftriaxone (*2*), a critically important antimicrobial agent for treating invasive salmonellosis in children (*3*).

Studies suggest that dairy cattle are a major US reservoir for MDR *S*. Newport (*4*–*6*). However, data documenting the prevalence of MDR *S.* Newport among infected human case-patients in dairy-intensive states are limited. To assess the prevalence of resistance among *S*. Newport isolates in Wisconsin, which in 2002 had the greatest density of milk cows in the United States (*7*), we evaluated antimicrobial susceptibility data from *S.* Newport infections among Wisconsin case-patients during 2003–2005. We also compared information on potential exposures for case-patients with Newport-MDRAmpC and susceptible infections.

## The Study

Surveillance data were electronically compiled for laboratory-confirmed *S.* Newport infections among Wisconsin residents with illness onsets from January 1, 2003, through December 31, 2005. Providers and local health departments reported hospitalization status; travel history; and exposure to raw milk, cattle, horses, reptiles, and dead animals. The study population included case-patients whose isolates were tested for antimicrobial drug susceptibility at the Wisconsin State Laboratory of Hygiene. Identification and susceptibility testing were conducted on isolates from stool, urine, and blood samples.

Serotype identification was performed according to the Kauffmann-White scheme (*8*). Slide and tube agglutination were used for identification of O (somatic) and H (flagellar) antigens, respectively. All isolates were tested for susceptibility to ampicillin, amoxicillin-clavulanic acid, cefoxitin, ceftriaxone, cephalothin, chloramphenicol, ciprofloxacin, gentamicin, kanamycin, nalidixic acid, streptomycin, sulfisoxazole, tetracycline, and trimethoprim-sulfamethoxazole, by using the Kirby-Bauer disk diffusion method. Results were interpreted according to Clinical and Laboratory Standards Institute (CLSI) guidelines (*9*). Antimicrobial agents were categorized into CLSI antimicrobial subclasses, and each isolate was assigned to >1 categories according to its antimicrobial resistance phenotype and the number of subclasses to which it was resistant (National Antimicrobial Resistance Monitoring System for Enteric Bacteria [NARMS], pers. comm.; Table 1). Pansusceptible isolates were defined as isolates that had no detected antimicrobial drug resistance. Because isolates were not tested for ceftiofur resistance, our definition of Newport-MDRAmpC did not include resistance to this drug.

**Table 1:** Antimicrobial drug resistance patterns of human *Salmonella* Newport isolates among case-patients*

Resistant to	Frequency (%)	
	Wisconsin (n = 268), 2003–2005	Rest of United States (n = 402), 2003–2004
None detected	95 (35)	317 (79)
>1 CLSI subclass†	173 (65)	85 (21)
>2 CLSI subclasses	150 (56)	81 (20)
>3 CLSI subclasses	150 (56)	77 (19)
>4 CLSI subclasses	150 (56)	74 (18)
>5 CLSI subclasses	146 (55)	71 (18)
At least ACSSuT‡	139 (52)	69 (17)
At least ACSuTm§	7 (3)	4 (1)
At least MDRAmpC¶	137 (51)	68 (17)
Quinolone and cephalosporin (third generation)#	5 (2)**	2 (0.5)

*Based on data from the National Antimicrobial Resistance Monitoring System for Enteric Bacteria. †CLSI, Clinical and Laboratory Standards Institute. Subclasses included aminoglycosides (kanamycin, gentamicin, streptomycin), aminopenicillins (ampicillin), β-lactamase inhibitor combinations (amoxicillin-clavulanic acid), first-generation cephalosporins (cephalothin), third-generation cephalosporins (ceftriaxone), cephamycins (cefoxitin), folate pathway inhibitors (trimethoprim-sulfamethoxazole), phenicols (chloramphenicol), quinolones (nalidixic acid, ciprofloxacin), sulfonamides (sulfisoxazole), and tetracyclines (tetracycline). ‡ACSSuT, ampicillin, chloramphenicol, streptomycin, sulfamethoxazole/sulfisoxazole, tetracycline. §ACSuTm, ampicillin, chloramphenicol, trimethoprim-sulfamethoxazole. ¶At least drugs to which MDRAmpC is resistant: chloramphenicol, streptomycin, trimethoprim-sulfamethoxazole, sulfisoxazole, tetracycline, amoxicillin-clavulanic acid, ampicillin, cefoxitin, cephalothin, and ceftriaxone. Note: the Wisconsin State Laboratory of Hygiene does not routinely test *Salmonella* isolates for resistance to ceftiofur, a third-generation cephalosporin that is related to ceftriaxone. #Resistant to ciprofloxacin and/or nalidixic acid, and ceftriaxone. **1 isolate in this category was also MDRAmpC.

The prevalence of each type of resistance among *S.* Newport isolates from Wisconsin case-patients was compared with that reported elsewhere in the United States, by using 2003 and preliminary 2004 NARMS data. Data were analyzed by using Epi Info 2002, version 3.3.2 (Centers for Disease Control and Prevention, Atlanta, GA, USA); to assess associations between antimicrobial resistance and reported exposures, odds ratios and Mantel-Haenszel and Fisher exact 2-tailed p values were calculated where appropriate.

Serotyping and antimicrobial drug susceptibility testing were conducted on *S.* Newport isolates from 268 case-patients. Median age was 34 years (range <1–96 years); of 267 case-patients for whom sex was reported, 57% were female. Resistance patterns are provided in Table 1. Among the 5 (2%) quinolone-resistant isolates (2 resistant to nalidixic acid and ciprofloxacin, 2 resistant to nalidixic acid only, and 1 resistant to ciprofloxacin only), 4 were ceftriaxone resistant and 1 was MDRAmpC resistant. The frequencies of antimicrobial drug resistance among Wisconsin *S.* Newport isolates were substantially greater for all resistance subgroups than frequencies reported elsewhere in the United States during 2003 and 2004 (NARMS, pers. comm.; Table 1; Figure).

**Figure:**
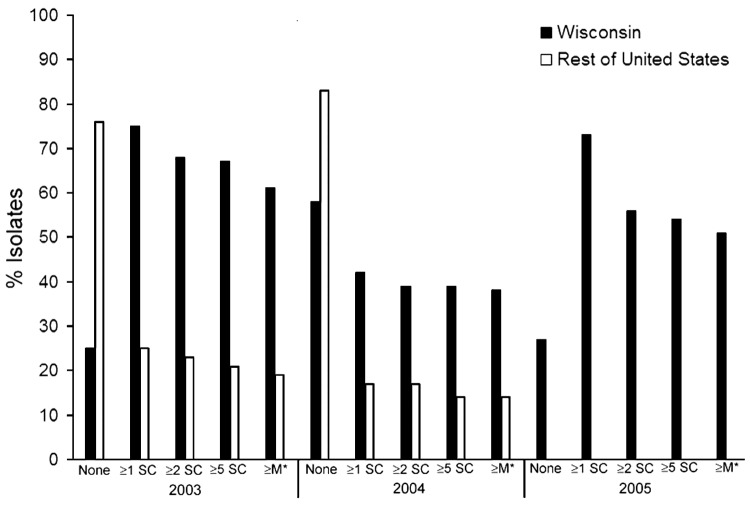
Antimicrobial drug resistance patterns of human *Salmonella* Newport isolates from Wisconsin (2003–2005) and elsewhere in the United States (2003–2004), based on data provided by the National Antimicrobial Resistance Monitoring System for Enteric Bacteria (NARMS). 2005 NARMS data were not available at the time of publication of this report. Antimicrobial subclasses are as defined by the Clinical and Laboratory Standards Institute (*9*). SC, subclass; M*, MDRAmpC.

Of 194 case-patients for whom hospitalization status was reported, 46 (24%) had been hospitalized. Of case-patients with Newport-MDRAmpC and pansusceptible infections, similar proportions were hospitalized (26% and 24%, respectively) and had known hospitalization status (72% and 73%, respectively). Two case-patients died: an 84-year-old woman and a 37-year-old man for whom salmonellosis was not considered the probable cause of death. The 2 associated isolates were pansusceptible.

Persons infected with Newport-MDRAmpC were significantly more likely than persons infected with pansusceptible *S.* Newport to be male and to have had contact with cattle, to have drunk unpasteurized milk, and to live on or have visited a farm or petting zoo (Table 2). Reported exposure to reptiles was significantly associated with pansusceptible infection (Table 2). No association was found between hospitalization and resistance (odds ratio [OR] 1.09, p = 0.81).

**Table 2:** Association between reported demographic and exposure variables and *Salmonella* Newport-MDRAmpC infections in Wisconsin case-patients, 2003–2005*

Variable†	Infection, n (%)		Odds ratio	p value
	MDRAmpC‡ (n = 137)	Pansusceptible (n = 95)		
Male	71(52)	30 (32)	2.33	0.002§
Contact with cattle	20 (15)	0	UD	0.0001§
Farm residence or farm or petting zoo visit¶	14 (10)	0	UD	0.001§
Consumption of raw milk	10 (7)	0	UD	0.006#
Contact with horses	2 (2)	0	UD	0.514#
Foreign travel	0 (0)	1 (1)	0	0.409#
Contact with dead animal	1 (7)	0	UD	1.000#
Contact with pet reptile	0 (0)	7 (7)	0	0.002#

*Salmonella Newport–multidrug-resistant AmpC (MDRAmpC) is resistant to at least chloramphenicol, streptomycin, sulfamethoxazole/sulfisoxazole, tetracycline, amoxicillin-clavulanic acid, ampicillin, cefoxitin, ceftiofur, and cephalothin and shows decreased susceptibility to ceftriaxone. Table includes case-patients with Newport-MDRAmpC and pansusceptible infections only. UD, undefined. †A specific exposure period was not assessed except for travel. Other exposures reported for case-patients included eating raw ground beef (1 MDRAmpC), eating raw cookie dough (1 MDRAmpC and 1 pansusceptible), preparing a raw chicken pet diet (1 pansusceptible), contact with an ill family member (1 MDRAmpC and 1 pansusceptible), and attending a pig roast (6 MDRAmpC and 1 pansusceptible). ‡At least MDRAmpC resistant. §Mantel-Haenszel χ^2^. ¶Exposure to farms and petting zoos was not explicitly assessed by the case reporting form. In all, 14 case-patients reported this exposure; all associated isolates were Newport-MDRAmpC. #Fisher exact test.

## Conclusions

We describe a substantially greater prevalence of MDRAmpC resistance among Wisconsin case-patients with *S.* Newport infections that occurred during 2003–2005, compared with data reported elsewhere in the United States (NARMS, personal communication, 2007). This finding is of particular concern because Newport-MDRAmpC exhibits decreased susceptibility to ceftriaxone, a third-generation cephalosporin that is the treatment of choice for invasive salmonellosis in children (*3*). Additionally, because the *bla*_CMY-2_ gene that confers ceftriaxone resistance in Newport-MDRAmpC is located on a plasmid that was readily transferred between *Escherichia coli* in laboratory assays (*10*), propagation of Newport-MDRAmpC could increase the spread of CMY-2 plasmids to other bacteria.

Patients with Newport-MDRAmpC infection were more likely than patients with pansusceptible infections to report contact with cattle, farms, and unpasteurized milk. These exposures are likely to be more common among patients with Newport-MDRAmpC infection than among the general Wisconsin population, which suggests that dairy cattle are an important reservoir for Newport-MDRAmpC. Increased prevalence of Newport-MDRAmpC in Wisconsin may be due to selective pressure from the use of antimicrobial drugs on dairy farms (*1*), particularly ceftiofur, an extended-generation cephalosporin closely related to ceftriaxone (which is commonly used in cattle) (*11*). Clonally and independently acquired CMY-2–associated ceftiofur resistance has been identified among *Salmonella* strains isolated from dairy farms (*12*).

Few published data are available on the prevalence of Newport-MDRAmpC in other dairy-intensive states. Minnesota, which in 2002 had the eighth-greatest density of milk cows in the United States (*7*), reported a significant increase in MDR *S.* Newport among human isolates during 1996–2003, including an increase in the percentage of isolates with decreased susceptibility to ceftriaxone (*13*). However, NARMS reported a similar increase in Newport-MDRAmpC prevalence nationally during 1998–2003 (*2*)*.* Analyses of unpublished data provided by the Minnesota Department of Health indicated that 22% of 147 human isolates tested had antimicrobial drug resistance profiles consistent with the Newport-MDRAmpC phenotype during 2003–2005; this prevalence is much lower than that among Wisconsin case-patients who were ill during the same period. Although differences in enteric disease surveillance could partially explain this discrepancy, Newport-MDRAmpC’s emergence in dairy cattle is likely to be associated with several factors.

Our findings underscore the need for intensive Newport-MDRAmpC surveillance in major dairy states. Efforts to promote the conservative and appropriate use of ceftiofur and other antimicrobial drugs in dairy cattle are indicated; they should be complemented by strategies to discourage transmission of MDR *Salmonella* among cattle, such as separating ill from parturient animals and disinfecting environmental niches (*14*). Furthermore, providers should be discouraged from prescribing antimicrobial drugs to patients with low-risk *Salmonella* infections (*15*), and public health messages should emphasize the importance of pasteurizing milk and cooking meat appropriately.
